# Disturbances, organisms and ecosystems: a global change perspective

**DOI:** 10.1002/ece3.505

**Published:** 2013-03-06

**Authors:** Jean-François Ponge

**Affiliations:** Muséum National d'Histoire Naturelle, CNRS UMR 71794 avenue du Petit-Château, Brunoy, 91800, France

**Keywords:** Anticipation, disturbances, ecosystems, evolution, global change, species

## Abstract

The present text exposes a theory of the role of disturbances in the assemblage and evolution of species within ecosystems, based principally, but not exclusively, on terrestrial ecosystems. Two groups of organisms, doted of contrasted strategies when faced with environmental disturbances, are presented, based on the classical r-K dichotomy, but enriched with more modern concepts from community and evolutionary ecology. Both groups participate in the assembly of known animal, plant, and microbial communities, but with different requirements about environmental fluctuations. The so-called “civilized” organisms are doted with efficient anticipatory mechanisms, allowing them to optimize from an energetic point of view their performances in a predictable environment (stable or fluctuating cyclically at the scale of life expectancy), and they developed advanced specializations in the course of evolutionary time. On the opposite side, the so-called “barbarians” are weakly efficient in a stable environment because they waste energy for foraging, growth, and reproduction, but they are well adapted to unpredictably changing conditions, in particular during major ecological crises. Both groups of organisms succeed or alternate each other in the course of spontaneous or geared successional processes, as well as in the course of evolution. The balance of “barbarians” against “civilized” strategies within communities is predicted to shift in favor of the first type under present-day anthropic pressure, exemplified among others by climate warming, land use change, pollution, and biological invasions.

## Introduction

Many studies showed that some species traits were better adapted than others to land use change, pollution, climate warming, or biological invasions (Fisker et al. 2011; Makkonen et al. 2011; Malmström 2012; Shine 2012). Such knowledge could be used to predict which species will survive, become extinct or will have to adapt during the present-day mass extinction (May 2010). Much research effort needs to be conducted in order to have a clear view of the future of plant, animal, and microbial communities face to increasing anthropic pressure (Berg et al. 2010). However, some testable predictions can be made, which is the scope of the present review, based on previous theoretical work already carried out by McArthur and Wilson (1963), Odum (1969) and Pianka (1970), enriched by more modern concepts and adapted to present-day threats to biodiversity in the context of global change. In a first step, anticipation will be considered as a key advantage or disadvantage according to predictability or unpredictability of the environment, respectively. In a second step, species traits, which contribute, or not, to anticipate disturbances will be examined in the light of evolutionary processes. At last, strategies by which organisms and communities can resist, or not, environmental hazards, will be discussed in the frame of global change.

In the present paper, “species” or “traits” are considered indifferently under the generic term of “organisms.” Clearly, traits and species do not evolve at the same rate (Janecka et al. 2012), are not selected or filtered in the same manner (Keddy 1992), and trait representation changes in the course of individual development (Coleman et al. 1994) and within metapopulations and species distribution ranges (Sun and Cheptou 2012).

### Anticipation: a key property of organisms and ecosystems in stable environments

Anticipation is the manner in which an organism or a community behaves in advance of a predictable event, whether favorable or unfavorable. Such a mechanism is advantageous, in terms of energetic cost and resource allocation (González-Gómez et al. 2011) in an environment dominated by cyclic processes. Biological clocks (Wang and Wang 2011) participate in this property, as well as all sensory, behavioral, and signaling systems allowing a being or a group of beings to live in harmony with the context (Soler et al. 2011). In this frame, any forwarded event cannot be considered as a disturbance, as the organism or the community reacts in adapted manner. Such adapted reaction norms have been selected and fixed in the genome (Roulin et al. 2011), or are epigenetically induced by the environment (Gorelick 2005). They can also stem from behavioral training, through the memorization of past experiences (González-Gómez et al. 2011), mimicry (Darst 2006), teaching or transmission of knowledge within a familial or social group (Fogarty et al. 2011), or even through clonal reproduction (Trewavas 2005).

Anticipation is progressively established in the course of ontogenesis, more especially when complex locomotory or sensory organs and coordinated nervous and hormonal systems are required (Capellán and Nicieza 2010).

If anticipation is well known in the physiology of organisms (Vitalini et al. 2007), this property has never been cited at the ecosystem or community level, although many processes ensuring the stability of late-successional ecosystems involve anticipation. Bernier and Ponge (1994) showed that the regeneration of mountain coniferous forests is ensured by the recovery of a complete earthworm community under mature trees, following collapse during the pole stage. The reconstitution of the earthworm community occurs well in advance of light arrival, associated with senescence and death of trees, the event previously thought to start regeneration. The complete earthworm community allows spruce recruitment, by creating soil conditions favorable to the establishment of a new generation of spruce (Ponge et al. 1998). Such mechanisms involve feedbacks between soil organisms and plants (Ponge 2013), in particular between the two dominant ecosystem engineers (trees and earthworms). There are serious reasons to believe that such interaction networks, ensuring the long-term stability of habitats, have been selected at the ecosystem level in the course of evolution (Williams and Lenton 2007).

Are all organisms equally efficient in terms of anticipation? The buildup of sensorimotor systems, associated with neuronal and/or chemical mechanisms, is a prerequisite, and needs time. Saving time is possible to a great extent for those organisms taking care of their offspring and/or of members of their group, thereby privileging teaching and mimicry over genetically wired information. Information, in the form of treatment and management of signals, is a key component of anticipation (Patten et al. 1997), but it can work efficiently and durably only in a predictable environment, i.e. in the absence of disturbance.

In the presence of a disturbance (in the restricted sense given to it here, i.e. an unpredictable event), some organisms, less efficient in terms of anticipation, may be favored if they are able to grow, reproduce, and interact with other members of the community in the absence of any refined knowledge of the environment or of the group to which they pertain. Organisms with a short lifespan, able to disperse and reproduce at a high rate, without any need of care and training of juveniles, as well as juveniles of anticipating organisms, will be favored by unpredictable events (Odum 1969; Pianka 1970). Generalists, too, i.e. organisms not specialized on a given environment or a given resource, are also favored by disturbances, when compared with specialists (Devictor et al. 2010; Poisot et al. 2012). At the ecosystem level, pioneer (early-successional) communities are ephemeral, of variable composition, and generally are replaced by more durable communities when and if the environment remains or becomes stable (Isermann 2011).

Stability of the environment may itself result from the development of communities, as in the case of old-growth forests and coral reefs: these ecosystems generate the conditions of their own stability, according to dynamic equilibria (Connell 1978). Ecosystem engineers (trees, earthworms, corals, among many others) play a decisive role in such dynamic equilibria (Bythell and Wild 2011). In the presence of a major disturbance falling out of the range of their tolerance range, durable ecosystems are replaced by ephemeral communities with a high capacity of colonization (Dudgeon et al. 2010).

### Can we predict the existence of strategies according to the disturbance regime?

Many species traits have been classified on the base of strategies performed by organisms to ensure their success within communities or in the course of evolution (both concepts are tightly related, see Metz et al. 2008). Table 1 sketches most well-known strategies of plant, animal, and microbial organisms, which all refer to some extent to the r-K continuum. Contrary to most of them, which are dual, The CSR triangle devised by Grime (1977) for plants distinguishes three strategies according to growth rate, site fertility, and competitive ability, thus mixing plant traits and environmental features in a common classificatory endeavor (Craine 2005). Similar continuum triangles of life-history strategies have also been proposed for animals (Winemiller 1992; Vila-Gispert et al. 2002). All these strategies consider stability of the environment as the driving force of their selection, as this was clearly explicit in the seminal work of Levins (1962), but rather implicit in most other studies listed in Table 1. By privileging this aspect and by assembling all traits which are related directly or indirectly to the stability of the environment, two categories of traits/organisms can be suggested. They are called “civilized” and “barbarians,” gathering features related to life history, ecological amplitude and evolution covered by previous classifications (Table 1). Traits classified as “civilized” are those, which allow anticipation of short-term variations in the environment (but not ecological crises), while “barbarian” traits allow life in unpredictable environments and survival of ecological crises. Vicarious categories listed in Table 1 are relative to particular aspects of life history, dispersal, and ecological specialization, which share many properties between them.

**Table 1:** Main strategies of ‘barbarian” and “civilized” organisms

Barbarians	Civilized		References
r-selected: numerous offspring, early reproduction, high mortality rate	K-selected: reduced offspring, late reproduction, weak mortality rate		Pianka 1970; Fierer et al. 2007;
Generalists: able to reproduce in a wide array of environments	Specialists: able to reproduce in a restricted array of environments		Levins 1968; Egas et al. 2004
Pioneers: colonizing new environments	Climax species: associated to terminal stages of an ecological succession		Odum 1969; Wehenkel et al. 2006;
Colonizers: short generation time, abundant offspring, high metabolic activity, resistant to pollution	Persisters: long generation time, reduced offspring, low metabolic activity, sensitive to pollution		Ettema and Bongers 1993; Li et al. 2005;
Search strategy by random movements: using coordinated, but never targetted movements	Search strategy by directional movements: using coordinated and targetted		Cain 1985; Armsworth and Roughgarden 2005;
Migrants: without any defined territory	Residents: living in a defined territory (maybe changing seasonally or annually: case of migratory birds and butterflies)		Austin 1970; Holt et al. 2011;
Juveniles and neotenic adults	Adults		Stearns 1976; Johansson et al. 2010;
Natural-selected: small-sized organisms, without sexual dimorphism, with high phenotypic plasticity	Sexual-selected: big-sized organisms, with sexual dimorphism, with poor phenotypic plasticity		McLain 1993; Prinzing et al. 2002a
Density-independence	Density-dependence		Nicholson 1933; Bårdsen and Tveraa 2012;
RuderaIs: fast-growing species inhabiting high-fertility, high-disturbance sites	Competitors: fast-growing species inhabiting high-fertility, low-disturbance sites	Stress-tolerators: slow-growing specie inhabiting low-fertility, low-disturbance sites	Grime 1977; Wilson and Lee 2000

Such a dichotomy between “barbarians” and “civilized” does not confer any superiority to one or the other category, according to the tenet by de Montaigne (1595) that barbarism and civilization are two facets of the same endeavor of mankind for surviving in the course of past and future history. The term “barbarians” might seem at first sight reminiscent of “Gengis Khan” species, an expression used by Pimm (1991) to designate struggling invaders. Here, the term of “barbarians” is used in a much broader sense, without focusing on potential species interactions and above all without any negative overtone.

Although widely employed in ecology, the term “strategy” implies a dichotomy between more or less exclusive combinations of life-history and behavioral traits, which can be thought at first sight incompatible with the original r-K continuum (Pianka 1970; Jones 1976). Surprisingly, few workers questioned the existence of a continuum, until Flegr (1997) simulated the impact of environmental fluctuations on mixed populations. He showed that simulated ecosystems switched in the course of time toward either one or the other strategy according to stochastic effects of stability or instability of the environment, r- and K-selection being mutually exclusive in the long-term. Similar conclusions were reached by Arditi et al. (2005) at community level.

Phylogenetic trees based on present species are the net result of macroevolutionary speciation and extinction processes (Morrow et al. 2003) and can be reconstructed using parsimonious methods such as cladistics (Hennig 1975). Species not far from the root of a phylogenetic tree share a majority of basal characters (undifferentiated), contrary to species located far from the root. We may consider that basal characters are present (today) in ancient species or species groups having crossed unpredictable ecological crises, i.e. “barbarians,” and derived characters in more recent species or species groups which did not experience such crises, i.e. “civilized.” According to these definitions, most “civilized” traits are prone to extinction, while most “barbarian” traits would survive a long time across lineages, or would reappear in variable environments (Colles et al. 2009). The idea that mass extinction acts under selective rules different from background extinction has been stressed by Jablonski (2008), pointing to the existence of a fruitful area at the interface of ecology and macroevolution, which still needs to be prospected before clear predictions could be made. Paleoecological studies associating species traits with extinction avoidance or sensitivity are scarce, because life history, physiology, and behavior of fossils are unknown, only ecological specialization and endemism being accessible to paleoecologists. It has been shown that most specialized organisms and endemic species or groups, here classified as “civilized” (Table 1), suffered more from mass extinction than poorly specialized, cosmopolitan species, here classified as “barbarian,” in the course of past ecological crises. This was the case, among many others, for foraminifers at the Cretaceous-Tertiary boundary (Keller et al. 2002) and brachiopods during the End-Permian mass extinction (Rodland and Bottjer 2001). Similar examples can be found in present-day temperature-driven extinction scenarios (Schippers et al. 2011).

We showed that “barbarians” privilege production by wasting energy for growth and reproduction and “civilized” privilege information by channeling energy on fine tuning. Then a trade-off is possible between two facets of adaptive cost, called “evolutionary cost” (dominant in “civilized”) and “energetic cost” (dominant in “barbarians”). Evolutionary cost is here defined as the number of evolutionary steps (favorable mutations and/or epigenetic events) needed to develop and perform an organizational model in a given environment. Energetic cost is here defined as the amount of energy necessary for survival and reproduction (foraging, avoidance, dispersal, mating, etc.…), which is constrained by food supply and temperature in a given environment. Demonstration of such a trade-off between long-term (evolutionary) and short-term (energetic) costs is difficult to achieve, but a recent study by Bekaert et al. (2012) showed that energetic limitations contributed to fitness costs of glucosinolates, a phylogenetically sensitive class of compounds produced by Brassicales, used here as a proxy of the evolution of herbivory resistance in *Arabidopsis thaliana*.

Each neuronal and/or hormonal network, needed to reduce the energetic cost of an organism, has an evolutionary cost (Niven and Laughlin 2008). Thereby, long-term stability of the environment is a prerequisite for the evolution of complex functional networks within a community (Vermeij 2012), pointing to (evolutionary) time as a constraint. The complication of organizational templates along lineages is often accompanied by an increase in size, as a result of species interactions (Vermeij 2012). This evolutionary arms race stems in deadlocks, often linked to gigantism and extreme specialization (Myers 1996). Evolutionary stasis is followed by abrupt changes, modifying the “groundplan” to create new lineages starting from “young”, little specialized (dedifferentiated, often neotenic) organisms (Futuyma and Moreno 1988). These newly created lineages are very dynamic from an evolutionary point of view, because of short life cycles and a high ability to colonize new environments (Salzburger et al. 2005). Evolutionary cycles of stability (coordinated stasis) alternating with intense radiation (DiMichele et al. 2004) are similar to successions observed at ecosystem level (Odum 1969). They suppose the existence of a major constraint imposing a correlation between physiology, shape, demography, and dispersal (Reed et al. 2010). Such a correlated response in the evolution of phenotypic plasticity (Scheiner 2002) implies a very limited number of possible (viable) cases, i.e. strategies, which have been detected by simulating virtual communities (Goudard and Loreau 2008). This was experimentally proven to occur in microbial communities (Williams and Lenton 2007).

**Figure 1:**
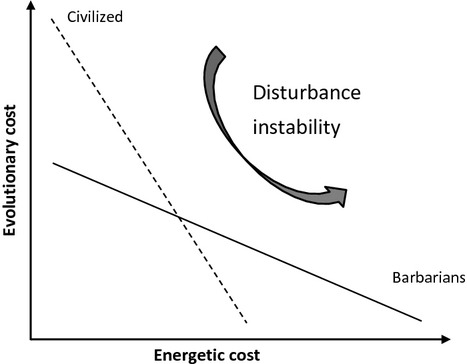
Evolutionary and energetic costs favor differently “barbarians” and “civilized” organisms, disturbances and environmental instability increase energy availability and decrease time to adapt.

Figure 1 explains in a scheme how constraints in energy and in time generate the existence of two non-exclusive strategies. Instability of the environment, above a given threshold making it unpredictable to organisms, generates a pressure making the “time” constraint dominant above the “energy” constraint. Only pre-adapted species (Afanasjeva 2010), with a wide tolerance breadth (here called “barbarians”) will subsist, possibly using epigenetic modifications induced by the environment (Angers et al. 2010) in addition to their innate phenotypic plasticity (Reed et al. 2010). On the contrary, when environmental stability recovers for a long period (Marcotte 1999), or when species find stable refuges (Dzik 1999), organisms using their energy for finely tuning with the environment (“civilized”) will take over, replacing “barbarians” (Wilson and Yoshimura 1994).

Although focus has been made above on abiotic environmental conditions, it must be clear that many biotic influences affect traits and/or organisms and their capacity to tolerate disturbances. The example of host–parasite interactions is particularly demonstrative in this respect. The parasite is a specialized organism, which in its way of life is associated with its host by many anticipatory mechanisms, allowing it to maintain viable populations (Tinsley 2004). When the parasite prevents (and thus anticipates) the death of its host by lowering its infectious potential below a threshold of tolerance (Duerr et al. 2004), it clearly belongs to the “civilized” category. More generally, all density-dependent processes, whether negative or positive (including the “Allee effect”), belong to the “civilized” category (Bårdsen and Tveraa 2012). The more an organism will be specialized on a host or a food resource or on conspecifics, the more it will be sensitive to any disturbance affecting the organism(s) on which it depends for its survival and reproduction (Montoya et al. 2009). Density-independent processes escape to such biotic control and thus rather belong to the “barbarian” category, associated with unpredictability (Sinclair and Pech 1996).

### “Barbarians” and “civilized” in a changing world: who will win and why?

**Figure 2:**
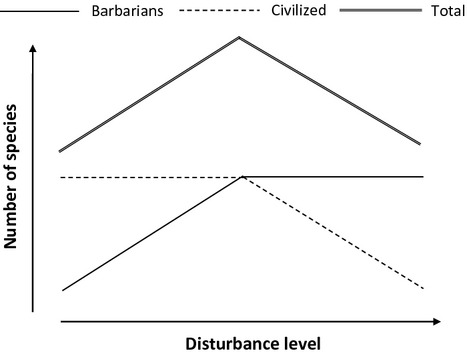
The “intermediate disturbance hypothesis” explained by the balance between “barbarians” and “civilized” species.

In the presence of environmental instability, “civilized” organisms are at disadvantage compared with “barbarians”, the contrary when conditions become stable again. However, “barbarians” and “civilized” may cohabit within the same community and perform complementary functions (Wilson and Yoshimura 1994) inasmuch as conditions are not those of a major ecological crisis affecting all biotopes present in a given region of the world. Examples of major ecological crises are great glaciations of the Pleistocene, K-T boundary and Permian-Triassic extinctions (Raup 1986). Both categories are just in balance according to the disturbance regime, along a continuum from most to least stable environments (Fig. 2). At an intermediate level of disturbance biodiversity is maximized because it is the ultimate level at which “barbarians” and “civilized” may cohabit within the limits of regional pools (Lessard et al. 2012), immigration waves (Esther et al. 2008) and species interactions (Mason et al. 2008). The fact that intermediate levels of disturbance (Molino and Sabatier 2001) and intermediate stages of succession (Isermann 2011) are favorable to local biodiversity can thus been explained by other hypotheses than resource limitation and competitive exclusion (Connell 1978).

Several aspects of global change have been selected for the following discussion: climate warming, pollution, land use change, biological invasions, and the registered (although still debated) increase in the amplitude and frequency of climatic and geologic catastrophes.

Climate warming, in particular the rapid temperature increase recorded over the last 40–50 years (Thompson et al. 2008), often associated with increased climate instability (Canale and Henry 2010), generates shifts in the distribution of plants and animals over large areas of the world (Thomas 2010). Within the same time lapse, many habitats collapsed (Clarke et al. 2007) or became fragmented (Cormont et al. 2012). Species with a high rate of phenotypic plasticity (Canale et al. 2011) and genetic diversity (Hoffmann and Willi 2008) are favored by climate warming. Species able to disperse and grow rapidly (Makkonen et al. 2011) as well as infectious diseases (Mouritsen et al. 2005) increase, and plant phenology changes in favor of earlier reproduction (Post et al. 2008). All these shifts in species traits point to an advantage given to “barbarian” over “civilized” traits. “Barbarians” are thus thought to increase until a new equilibrium will be reached, which would turn to an advantage given to “civilized” traits, species or phenotypes.

It has been observed that species with a wide geographic range are favored by climate warming (Arribas et al. 2012), as this occurred in the course of past ecological crises (Jablonski 2005). Fonty et al. (2009) showed that all plant life-forms and dispersal modes recorded in a tropical ecotone were affected by local extinctions, in inverse proportion to their rate of presence. Thus, commonness seems to be selected against rarity in the course of extinction events (Reinhardt et al. 2005), another clue to an advantage given to species commonly classified as “generalists” (Remold 2012) and/or with a high dispersal ability (Tedesco et al. 2012) against “specialists” and/or with a low dispersal ability, i.e. on “barbarians” against “civilized”. However, global climate warming is often accompanied by severe drought events which impact dramatically more sensitive environments, such as tropical rainforests (Wright and Calderón 2006), and the more when these forests are disturbed by logging (Curran et al. 1999). In such cases, the stress component (drought) may overwhelm the disturbance component of environmental change (Condit et al. 1995). Selection shifts then in favor of stress-tolerance traits (Read and Stokes 2006), belonging to the “civilized” strategy, rather than of disturbance traits (Jonsson and Esseen 1998), which belong to the “barbarian” strategy.

The effects of pollution and acidification of soil and atmosphere have been studied intensively during the last 40 years. Field studies showed severe disruptions in the composition of communities (Syrek et al. 2006), and irreversible collapses in ecosystem functioning, stemming in organic matter accumulation (Gillet and Ponge 2002). Hågvar (1990) elaborated an elegant hypothesis, highlighting behavioral choice and competition as driving forces of the response of species to soil acidity. But which life-history and dispersal traits are favored by pollution? Sexual reproduction seems to be favored over clonal reproduction in severely polluted conditions (Niklasson et al. 2000), and it has been suspected that some (apparently healthy) communities of polluted areas are maintained by high immigration rates compensating for high mortality (Møller et al. 2012). Both features are typical of unspecialized species privileging phenotypic variety (Poisot et al. 2012), with a high rate of nondirectional random movements to unfavorable places (Auclerc et al. 2010). Comparisons between polluted and nonpolluted areas show that species living in polluted environments are smaller, reproduce earlier and have a shorter lifespan (Prinzing et al. 2002b). All these characters point to many advantages given to “barbarian” over “civilized” traits. However, some studies did not reveal the same trends in aquatic faunas (Postma et al. 1995). This questions pollution as a stress or a press disturbance (Arens and West 2008), for which species can develop special mechanisms for tolerance in a stable environment (Janssens et al. 2009), i.e. of “civilized” type, or as a pulse disturbance, for which species survival must rely on rapid growth, reproduction and active dispersal, i.e. “barbarian” type.

Landuse change and fragmentation are known to favor species displaying a low level of specialization (Barbaro and Van Halder 2009) and/or moving easily through a heterogeneous landscape (Malmström 2012). Both groups of trait attributes belong clearly to the “barbarian” category. Similar shifts in trait representation were observed following the destruction of coral reefs (Feary 2007). However, the case of butterflies deserves a special attention, as some species with a high degree of specialization and efficient directional movements are able to withstand fragmentation, at least up to a certain threshold (Bergerot et al. 2012). These species, which are tolerant of fragmentation despite of their “civilized” characters, are in fact adapted to fragmented habitats in a stable landscape (Schtickzelle et al. 2007). This is also the case of mass migrations through heterogeneous landscapes observed in some collembolan species (Gauer 1997). Such heterogeneous environments can be considered stable at landscape scale (although changing in the course of time at local scale) and thus are predicted to select for “civilized” traits.

Biological invasions are still imperfectly understood from the point of view of threats to biodiversity and community change. In particular, changes in trait distribution of affected communities are poorly documented (Westley 2011). In the plant kingdom, invasive species are characterized by a high production rate (McAlpine et al. 2008), often associated with high phenotypic plasticity and efficient dispersal mechanisms (Thiébaut 2007). Most recorded effects of biological invasions are negative effects, such as habitat loss (Lichstein et al. 2004) and increased nutrient availability (Vitousek et al. 1987). However, high litter input and the ease with which invasive plants exploit soil nutrient resources may also help create new habitats (Maurel et al. 2010). In the animal kingdom, invasive species displace equilibria within communities either by predation or by competing with indigenous species, at least in initial phases of invasion (Morrison 2002), and they benefit from disturbances caused by human activities (Chauvel et al. 1999). They may also change profoundly existing habitats, as in the case of ecosystem engineers such as earthworms (Eisenhauer et al. 2009). In all these cases, indigenous specialists (Almeida-Neto et al. 2010), or species unable to find rapidly safe refuges (Urban and Titus 2010) are at disadvantage, pointing in turn to a selection in favor of “barbarians.” Invasive species themselves can be classified as “barbarians,” given their high investment in growth and reproduction, and more generally their versatility (Prinzing et al. 2002a).

That unprecedented recurrent catastrophes can be attributed to global change, in particular climate warming, is often advocated although still debated (Changnon 2009). If true, this gives strength to several predictions of the Gaia model (Kleidon 2010). Although poorly documented from an adaptive point of view, present-day catastrophic events such as earthquakes, tsunamis or storms destroy habitats to the same extent and with the same rapidity as fires, pollution, deforestation, mining activities, etc. As a consequence, traits associated with catastrophic “natural” disturbances do not differ from those associated with environmental hazards directly caused by human activities: “barbarian” traits (see Table 1) are advantageous, mostly because they do not rely on anticipation for ensuring survival and reproduction of species. Paleontological studies showed that “barbarian” traits were associated with organisms benefiting from mass extinctions which occurred at Permian-Triassic and Cretaceous-Paleogene (K-T) boundaries (Fawcett et al. 2009).

## Conclusions

The importance of anticipatory processes occurring in the adaptation of organisms to stable or unstable environments has been stressed. Two categories of traits or of organisms can be identified, called “civilized” and “barbarians,” according to advantage or disadvantage of anticipating disturbing events, respectively. This corresponds to the classical r-K dichotomy (Pianka 1970), here enlarged to several other commonly used classifications, such as “generalists” and “specialists” (see Table 1 for further details). It allows predicting which series of traits are or will be favored by present-day global change and associated disturbances. From a practical point of view, traits can be classified into “barbarians” and “civilized” on the base of properties which define them (absence or presence of anticipatory mechanisms, respectively) or proxies of these properties listed in Table 1. Changes in trait representation are expected to occur first, as a given species can shift rapidly from a strategy to another, as this has been shown to occur in bird species once classified as “specialists” (Barnagaud et al. 2011; see also review by Colles et al. 2009). The place taken by “barbarian” traits within communities, whether due to species replacement or to adaptation (selection or phenotypic plasticity), is thought to increase dramatically, at least until a new equilibrium state, if any, will be reached in a more or less near future (Jump and Peñuelas 2005). This increase does not necessarily cope with a corresponding decrease in “civilized” traits or species except when a threshold of tolerance is attained in the “civilized” category (see Fig. 2). Present-day collapses in global biodiversity (Laurance et al. 2012) seem to indicate that this threshold has been reached (Barnosky et al. 2012).
